# DREAMM: a web-based server for drugging protein-membrane interfaces as a novel workflow for targeted drug design

**DOI:** 10.1093/bioinformatics/btac680

**Published:** 2022-11-10

**Authors:** Alexios Chatzigoulas, Zoe Cournia

**Affiliations:** Biomedical Research Foundation, Academy of Athens, Athens 11527, Greece; Department of Informatics and Telecommunications, National and Kapodistrian University of Athens, Athens 15784, Greece; Biomedical Research Foundation, Academy of Athens, Athens 11527, Greece; Department of Informatics and Telecommunications, National and Kapodistrian University of Athens, Athens 15784, Greece

## Abstract

**Summary:**

The allosteric modulation of peripheral membrane proteins (PMPs) by targeting protein-membrane interactions with drug-like molecules represents a new promising therapeutic strategy for proteins currently considered undruggable. However, the accessibility of protein-membrane interfaces by small molecules has been so far unexplored, possibly due to the complexity of the interface, the limited protein-membrane structural information and the lack of computational workflows to study it. Herein, we present a pipeline for drugging protein-membrane interfaces using the DREAMM (Drugging pRotein mEmbrAne Machine learning Method) web server. DREAMM works in the back end with a fast and robust ensemble machine learning algorithm for identifying protein-membrane interfaces of PMPs. Additionally, DREAMM also identifies binding pockets in the vicinity of the predicted membrane-penetrating amino acids in protein conformational ensembles provided by the user or generated within DREAMM.

**Availability and implementation:**

DREAMM web server is accessible via https://dreamm.ni4os.eu.

**Supplementary information:**

Supplementary data are available at *Bioinformatics* online.

## 1 Introduction

Peripheral membrane proteins (PMPs) have emerged as promising therapeutic targets for several diseases, such as tuberculosis and cancer (Boes *et al.*, 2021). Moreover, the recent patent cliff has generated an increased interest in alternative drug design strategies over the past few years, especially focusing on proteins that have been considered undruggable up to now (Chatzigoulas and Cournia, 2021). Drugging protein-membrane interfaces of PMPs is a new promising therapeutic strategy for orthosteric inhibition or allosteric modulation of PMPs presumed undruggable (Cournia and Chatzigoulas, 2020; Segers *et al.*, 2007; Spiegel *et al.*, 2004). This drug design strategy has been so far overlooked possibly due to the complexity of the interface, the limited number of protein-membrane binding structural studies, and the lack of a suitable theoretical background combined with efficient *in silico* workflows. Nevertheless, studies targeting the protein-membrane interfaces support the fact that these interfaces are druggable, and targeted drug design can be implemented for PMPs involved in deregulated cellular pathways and disease (Chen *et al.*, 2015; Li and Buck, 2020; Liu *et al.*, 2010; Nawrotek *et al.*, 2019; Nicolaes *et al.*, 2014; Segers *et al.*, 2007; Spiegel *et al.*, 2004).

**Fig. 1.**
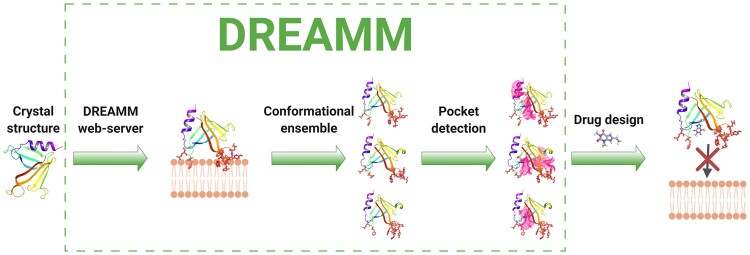
A schematic workflow of the DREAMM web server

Several computational tools and web servers for predicting protein-membrane interfaces, membrane domains and lipid-binding sites have been designed in the past (Bhardwaj *et al.*, 2006; Fuglebakk and Reuter, 2018; Kufareva *et al.*, 2014; Lomize *et al.*, 2012; Nastou *et al.*, 2016; Scott *et al.*, 2006; Sharikov *et al.*, 2008); however, these are limited to only predicting protein-membrane interfaces and domains and they are not integrated into an efficient peripheral membrane drug discovery workflow. Herein, the protein-membrane interface prediction is followed by an automated drug design workflow that accepts as input protein conformational ensembles and then, searches for binding sites in the protein-membrane interface of each conformer (Fig. 1). These binding sites are provided to the user to continue with docking studies for targeting the protein-membrane interface. DREAMM is offered as a free and open to all web server without login requirements which can be accessed at https://dreamm.ni4os.eu.

## 2 Materials and methods

To predict protein-membrane interfaces, we designed a novel ensemble machine learning classifier for predicting membrane-penetrating amino acids (Chatzigoulas and Cournia, 2022). A summary of our ensemble classifier model is provided in the Supplementary information. Following the prediction of protein-membrane interfaces, DREAMM also provides the option to predict binding cavities in the vicinity of the predicted membrane-penetrating amino acids using the open-source binding site detection software P2Rank (Krivák and Hoksza, 2018). To account for the dynamic nature of proteins, DREAMM applies P2Rank in conformational ensembles obtained by structural biology experiments or by molecular dynamics (MD) simulations; these structures can be provided by the user. In case a conformational ensemble is unavailable, DREAMM generates protein conformations using the distance geometry-based method ExProSE (Greener *et al.*, 2017). Using ExProSE, DREAMM generates protein conformations using the default settings and a tolerance weighting factor (weighting of constraint tolerances for interactions) W_B_ = 0.4. With this tolerance weighting factor value, we expect to generate structures with a small root-mean-square deviation compared to the input structure (<3 Å), albeit still observing changes in the binding sites. It should be noted that ExProSE does not perform MD simulations to generate a conformational ensemble of protein configurations. A more accurate description of the conformational ensemble can be achieved using MD simulations coupled with enhanced sampling techniques such as metadynamics, replica exchange and umbrella sampling (Barducci *et al.*, 2011). DREAMM performs the protein-membrane interface prediction on the first structure of the provided conformational ensemble; in the case of ExProSE usage, the prediction is performed in the initial Protein Data Bank (PDB) structure. Thus, in case a conformational change is necessary to place membrane-penetrating amino acids towards the membrane, this conformation (if available) should be provided as the first structure in the ensemble because the predictions depend on structural information. In case the user uploads AlphaFold2 structures (Jumper *et al.*, 2021), these structure must be preprocessed to exclude low-confidence (pLDDT score < 70) unstructured regions such as the N- and C-termini (for more information see the user manual). All structures of the protein conformational ensemble are then used to predict binding cavities in the vicinity of the identified membrane-penetrating amino acids as described below. Protein conformations are prepared and aligned with High-Throughput Molecular Dynamics (HTMD) Python package (Doerr *et al.*, 2016) and then, P2Rank predicts binding pockets in each protein conformation. DREAMM discards predicted binding pockets further than 5 Å from the closest atom of the predicted membrane-penetrating amino acids and then produces 3D visualizations of the predicted membrane-penetrating amino acids and the aforementioned binding pockets using PyMOL (Schrödinger, 2015) scripts generated by P2Rank. Moreover, DREAMM performs a conservation analysis of amino acid sequences to assess the conservation and diversity of the predicted binding sites (Remmert *et al*., 2012). DREAMM also evaluates the binding sites implication in large-scale protein motions using the Essential Site Scanning Analysis method (Kaynak *et al*., 2020) (for more details see the Supplementary information). To assist interpretation of the results, the identified binding pockets are clustered based on their center coordinates using k-means (Lloyd, 1982). The number of clusters, *k*, is chosen based on the Silhouette coefficient method (Rousseeuw, 1987) utilizing the scikit-learn Python package (Pedregosa *et al.*, 2011). The results of clustering are output to the user providing analyses for each binding pocket, e.g. the number of protein conformations where the binding site is found, the highest P2Rank score and the corresponding protein conformation, the mean conservation score, etc. (for more information see the ‘Download results’ and ‘Results interpretation’ section in the user manual). More information about DREAMM methodology is provided in the user manual and the Supplementary information.

## 3 Web server

DREAMM web server is designed with the markup and web languages HTML, CSS and JavaScript in the front end, and the PHP programming language in the back end, providing a user-friendly interface. The functionality of DREAMM is straightforward. The users input the four-letter PDB code and the chain name of the protein and hits the ‘upload’ button, or alternatively, select and upload their own PDB structure. If a user chooses to generate conformational ensembles using ExProSE, up to 50 conformations may be generated. Calculations last from a few minutes up to a few hours depending on the size of the protein. Furthermore, a queuing system runs in the background of the web server, allowing two calculations to run simultaneously and avoiding job failures due to large workload. Finally, to avoid waiting on the screen for the results, a unique URL for each job is provided, which can be bookmarked and accessed at a later time. Subsequently, the results are provided to the user, indicating which (if any) amino acids are predicted to insert into the membrane, and are visualized in the web server with JSmol (Hanson *et al.*, 2013). A download button allows the user to download the results including binding pocket predictions, PyMOL visualizations, and information on all binding sites at the protein-membrane interfaces of all conformations along with the clustering results. More information is provided in the user manual and the video tutorial. This service was subsequently on-boarded in the European Open Science Cloud, https://dreamm.ni4os.eu/.

## 4 Use cases

The protein-membrane interface prediction function of DREAMM has been thoroughly tested as described in Refs. (Chatzigoulas and Cournia, 2022; Jiang *et al.*, 2022; Valenstein *et al*., 2022). Moreover, DREAMM was assessed on four recently crystallized conformations of the protein FakB1, whose W180 is an important amino acid for FakB1-membrane binding (Gullett *et al.*, 2022). In all four conformations, DREAMM predicts W180 and adjacent amino acids as membrane penetrating (Supplementary Table S1 and Supplementary Fig. S1).

To test the drug design pipeline of DREAMM, the binding site prediction module of DREAMM using the ‘protein conformational ensembles’ function was applied to the nuclear magnetic resonance structure of the PH domain of ceramide transfer protein [PDB ID: 2RSG (Sugiki *et al.*, 2012)]. All structures were prepared and aligned with HTMD, and then, P2Rank was used within DREAMM to predict binding sites. Two putative binding sites were identified in the vicinity of the predicted membrane-penetrating amino acids W33, N35, Y36, I37, G39 and W40. After clustering the binding pockets of all different conformations, a consensus binding site emerged in 19/20 conformations and a second binding site emerged in one conformation (Supplementary Table S2 and Supplementary Fig. S2). Rational drug design in the binding site with the best average P2Rank score, which is the consensus binding site, could be envisaged for modulating the ceramide transfer protein function (Chung *et al.*, 2021).

## Funding

This work was supported by the State Scholarships Foundation (ΙΚΥ) [MIS-5000432 to A.C.]; and the Hellenic Foundation for Research and Innovation (H.F.R.I.) [1780 to Z.C.]. We thank the European Union's Horizon 2020 European research infastructures, “National Initiatives for Open Science in Europe – NI4OS Europe” project (grant agrrement no. 857645) for providing us with a virtual machine, and a domain for our web server and onboarding this service to the European Open Science Cloud. We acknowledge computational time granted from the Greek Research & Technology Network (GRNET) in the National HPC facility – ARIS under project IDs pr008033_gpu/Mem-Surf and pr008033_thin/Mem-Surf.

*Conflict of Interest*: none declared.

## Supplementary Material

btac680_Supplementary_Data
