# Supplementary material for: DREAMM: a web-based server for drugging protein-membrane interfaces as a novel workflow for targeted drug design
Source: Bioinformatics. 2022 Nov 10;38(24):5449–51. doi: 10.1093/bioinformatics/btac680 (PMC9750117; doi:10.1093/bioinformatics/btac680)
Supplement: btac680_Supplementary_Data [file btac680_supplementary_data.zip › btac680_Supplementary_Data/OP-CBIO220690_AuthorCorr_AttachmentsFolder_DREAMM_SI[AU].pdf]

SUPPORTING INFORMATION

DREAMM: A web-based server for drugging protein-membrane interfaces as a novel workflow for targeted drug design

Alexios Chatzigoulas[1,2] and Zoe Cournia[1,2,*]

[1]Biomedical Research Foundation, Academy of Athens, 11527 Athens, Greece

[2]Department of Informatics and Telecommunications, National and Kapodistrian University of Athens, 15784 Athens, Greece

Methods

The ensemble classifier model for predicting membrane-penetrating amino acids

In Ref (1) we describe in detail our ensemble machine learning methodology and algorithm for predicting membrane-penetrating amino acids.

Briefly, as a dataset, we used 54 peripheral membrane proteins with known 3D structures and experimentally known membrane-penetrating amino acids retrieved after an extensive literature search. Therefore, we set up a binary classification problem to distinguish between the class of membrane-penetrating amino acids and the class of non-membrane-penetrating amino acids. We then split our dataset into training and validation sets and proceeded with data selection in the training set. We also assembled a test set of 11 peripheral membrane proteins with known 3D structures and experimentally known membrane binding regions. To ensure that the predictions are unbiased, we calculated the percentage of identical amino acids for all sequence pairs in all datasets by setting a sequence identity threshold of 40%, to guarantee that the validation and test sets are diverse compared to the training set.

Then, we extracted 92 physicochemical and biochemical amino acid features using DSSP for secondary structure definition (2), FreeSASA for the solvent-accessible surface area (3), MSMS for the amino acid and Cα depth (4), the Wimley-White whole-residue interface and octanol hydrophobicity scales (5, 6), PDB2PQR for the charges (7, 8), HHblits for the conservation score (9), PRODY for the squared fluctuations (10, 11), the number of nearby amino acids, and others. Furthermore, for each amino acid we calculated the mean values of the aforementioned features for neighboring amino acids within a cutoff distance of 7 Å. In addition, we extracted 2788 features with the ProtDCal tool (12), which calculates many thermodynamics, topographic, and property-based features. Then, we removed redundant and non-significant features resulting in 560 features in the end.

The next step was to train and optimize the hyperparameter space of 21 different machine learning classifiers and feed their predictions into meta-classifiers. We applied the voting classifier, which classifies a sample based on the majority of the predictions of the first-level classifiers and the stacking classifier, which trains a classifier on the predictions of the first-level classifiers to obtain the final prediction. For both meta-classifiers, we tested all possible combinations of the first-level classifiers to find the best combination of classifiers. To evaluate the meta-classifiers' results we used the validation set. The best meta-classifier model was the voting classifier of five first-level classifiers, achieving an F_1 score = 92% and MCC = 0.84 in the classification of membrane-penetrating or non-membrane-penetrating amino acids.

In addition, we tested the performance of our tool on an independent test set of 11 peripheral membrane proteins with known membrane-binding regions, but with unknown precise membrane-penetrating amino acids. As there are no specific experimental membrane-penetrating amino acids, we consider predictions located in the membrane-binding regions or at the membrane plane defined by these regions as true positives. Again, our model displayed high accuracy: precision score = 91%. For more information about the ensemble classifier model for predicting membrane-penetrating amino acids please refer to Ref. (1).

Binding site conservation analysis of amino acid sequences

To assess the conservation and diversity of the putative binding sites in the predicted protein-membrane interface, we perform a conservation analysis of amino acid sequences to identify whether this site is evolutionary conserved. DREAMM applies the homology detection software HHblits (9), which is based on pairwise comparison of profile hidden Markov models. HHblits generates a multiple sequence alignment (MSA) by searching the UniClust30 database (13), which is the clustered UniProtKB sequence database at the level of 30% pairwise sequence identity. To calculate the sequence profile of a protein sequence and the amino acid conservation score using HHBlits, DREAMM utilizes the getSequenceProfile Python wrapper of HTMD software (14) iterating through the Uniclust30 database three times in order to find remote homologues. The amino acid conservation score (which is between 0 and 1) is calculated from the amino acid frequencies in the columns of the MSA. Then, DREAMM calculates the mean conservation score of the amino acids in the vicinity of the binding sites, which are predicted utilizing the open-source binding site detection software P2Rank (15). For more information, please refer to the user manual of DREAMM, https://dreamm.ni4os.eu.

Analysis of the binding site implication in large-scale protein motions

To assess the possible functional role of the putative binding sites in the predicted protein-membrane interface, we evaluate their implication in large-scale protein motions. DREAMM applies the elastic network model-based methodology Essential Site Scanning Analysis (ESSA) (16) to find amino acids that alter the global dynamics upon ligand binding. To mimic ligand binding, ESSA increases the local elastic network density by adding all heavy atoms of the scanned amino acid and then it evaluates the percent shift in the eigenvalues of the first 10 normal modes. In the end, the *z*-score is calculated for each amino acid to build the ESSA profile. A *z*-score above 0.59 suggests that the examined amino acid is in the top quartile (top 25%) of the *z*-scores, meaning that it is an important amino acid for protein function. In Ref. (16) it is suggested that a binding pocket can be ranked based on the median *z*-score of the amino acids in the vicinity of the binding pocket. Similarly, in DREAMM we calculate the median ESSA *z*-score of the amino acids in the vicinity of the binding sites, which are predicted utilizing the open-source binding site detection software P2Rank (15). The binding sites in all protein conformations are clustered and the mean values of the median ESSA *z*-scores are provided to the user (see below).

Binding site clustering

In DREAMM, P2Rank predicts binding pockets in all protein conformations, as it is described in the main text. To assist the user to interpret the binding pocket prediction results, the binding sites in the vicinity of the predicted protein-membrane penetrating amino acids are clustered based on their center coordinates using k-means (17). The number of clusters, k, is chosen according to the Silhouette coefficient method (18) using the scikit-learn Python package (19) in the following range of k values:

$$k = [a, 3a]$$

where a is the number of binding sites found in the conformation with the most binding sites in the vicinity of the predicted membrane-penetrating amino acids.

If the number of binding sites in the conformation with the most binding sites is 1, then the Silhouette coefficient method cannot be applied and the mean-shift clustering algorithm (20) is utilized using a 4 Å bandwidth and the scikit-learn Python package (19).

Tables

Table S1. DREAMM predictions on three open form conformations and one closed form conformation of FakB1 protein (21).

	**FakB1(A121I)-Palmitate (open)**	**FakB1(A158L)-Myristate (open)**	**FakB1(A121I, A158L)-Palmitate (open)**	**FakB1(R173A)-Palmitate (closed)**
PDB ID	6MH9	6NM1	7SG3	7SCL
Membrane penetrating amino acids predicted by DREAMM	A177, W180, and L184	W180, V181, L184, and L185	W180 and L184	W180, G182, T183, L185, and K186

Table S2. Clustering of binding sites discovered by P2Rank in the NMR ensemble of 20 structures of the PH domain of the ceramide transfer protein (PDB ID: 2RSG (22)). Each row is a binding site in the vicinity of the predicted protein-membrane interface showing the number of conformations that the binding site is found, the neighboring predicted membrane-penetrating amino acids, the highest P2Rank score and the corresponding protein conformation, the mean distance between the predicted membrane-penetrating amino acid and the closest surface atom of the binding site, the mean distance between the predicted membrane-penetrating amino acid and the binding site center, the mean binding site conservation score, and the mean binding site ESSA z-score.

**#**	**# of conformations**	**Membrane-penetrating amino acids**	**Highest P2Rank score and #conformation**	**Mean distance from the binding site (Å)**	**Mean distance from the binding site center (Å)**	**Mean conservation score**	**Mean ESSA z-score**
**Binding site 1**	19	W33, N35, Y36, I37, G39, and W40	7.4<br>#6	0.6	7.6	0.22	0.55
**Binding site 2**	1	W33, N35, Y36, I37, G39, and W40	1.1<br>#6	0	3.4	0.04	-0.78

Figures

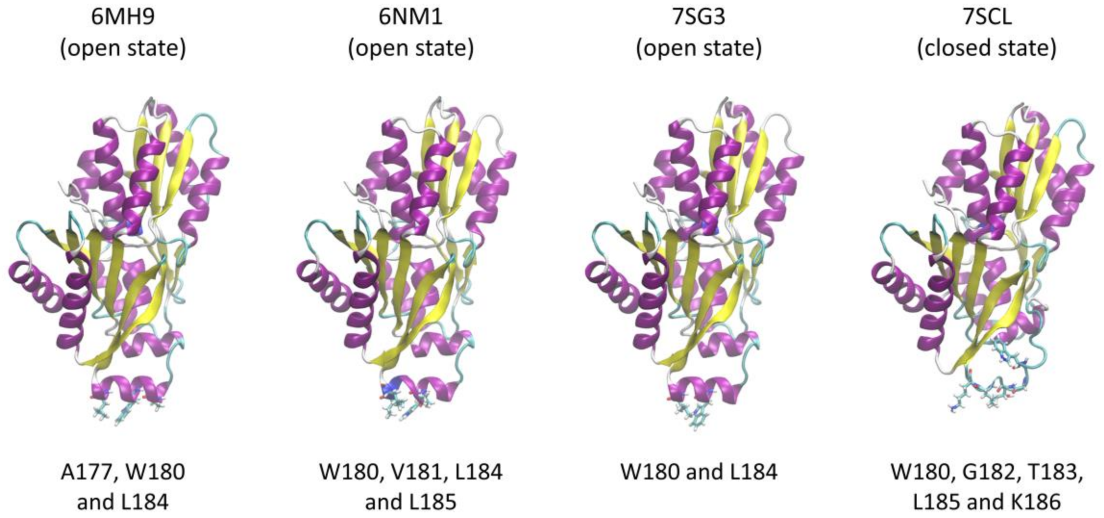

Figure S1. DREAMM predictions for the three conformations of the open form and one conformation of the closed form of FakB1. The predicted membrane-penetrating amino acids are shown in licorice format. The protein is colored based on the secondary structure. Magenta corresponds to α-helices, blue to 3_{10} helices, yellow to β-sheets, aqua corresponds to turns, and white to coils.

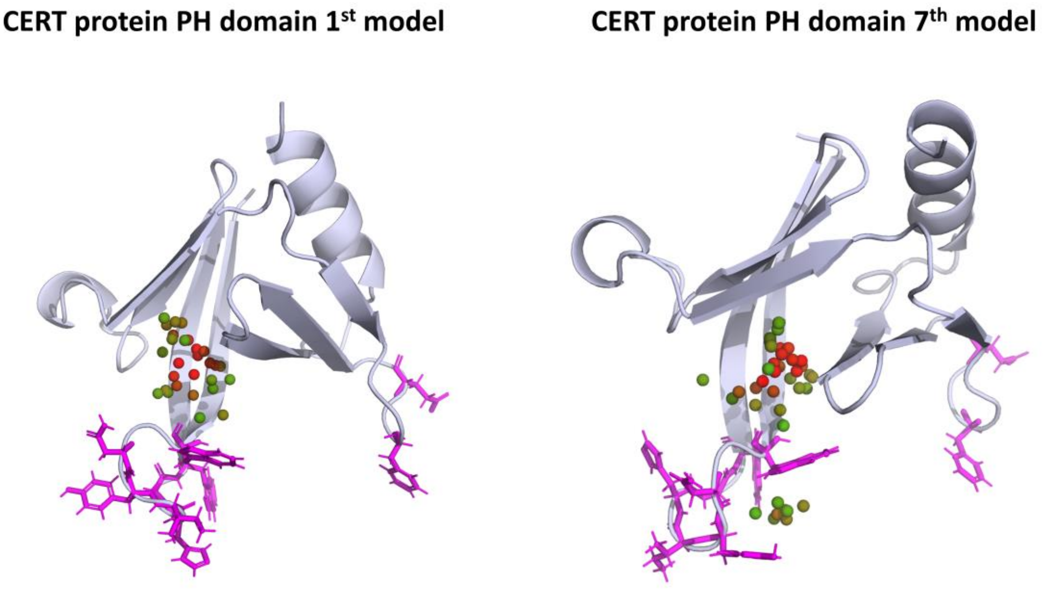

Figure S2. A PyMOL (23) visualizations of the first (left) and seventh (right) models of the NMR structure of the PH domain of ceramide transfer protein (PDB ID: 2RSG (22)) that were produced by the DREAMM. The binding sites are automatically predicted using P2Rank (15). The PyMOL script generated from P2Rank is automatically modified to display the predicted membrane-penetrating amino acids in purple and the binding sites within a distance of 5 Å from the closest atom of the predicted membrane-penetrating amino acids. In the first model (left) three binding sites were predicted with one of them being near the predicted membrane-penetrating amino acids and which is being displayed. In the seventh model (right) two binding sites were predicted both of them being within a distance of 5 Å from the predicted membrane-penetrating amino acids. For more information, please refer to DREAMM’s user manual.

References

1. Chatzigoulas A, Cournia Z. Predicting protein–membrane interfaces of peripheral membrane proteins using ensemble machine learning. Brief Bioinform. 2022;23(2):bbab518.
2. Kabsch W, Sander C. Dictionary of protein secondary structure: pattern recognition of hydrogen-bonded and geometrical features. Biopolymers. 1983;22(12):2577-637.
3. Mitternacht S. FreeSASA: An open source C library for solvent accessible surface area calculations. F1000Res. 2016;5:189.
4. Sanner MF, Olson AJ, Spehner JC. Reduced surface: an efficient way to compute molecular surfaces. Biopolymers. 1996;38(3):305-20.
5. Wimley WC, White SH. Experimentally determined hydrophobicity scale for proteins at membrane interfaces. Nat Struct Mol Biol. 1996;3(10):842-8.
6. Wimley WC, Creamer TP, White SH. Solvation energies of amino acid side chains and backbone in a family of host-guest pentapeptides. Biochemistry. 1996;35(16):5109-24.
7. Dolinsky TJ, Nielsen JE, McCammon JA, Baker NA. PDB2PQR: an automated pipeline for the setup of Poisson-Boltzmann electrostatics calculations. Nucleic Acids Res. 2004;32:W665-7.
8. Dolinsky TJ, Czodrowski P, Li H, Nielsen JE, Jensen JH, Klebe G, et al. PDB2PQR: expanding and upgrading automated preparation of biomolecular structures for molecular simulations. Nucleic Acids Res. 2007;35:W522-5.
9. Remmert M, Biegert A, Hauser A, Soding J. HHblits: lightning-fast iterative protein sequence searching by HMM-HMM alignment. Nat Methods. 2011;9(2):173-5.
10. Bakan A, Meireles LM, Bahar I. ProDy: protein dynamics inferred from theory and experiments. Bioinformatics. 2011;27(11):1575-7.
11. Bakan A, Dutta A, Mao W, Liu Y, Chennubhotla C, Lezon TR, et al. Evol and ProDy for bridging protein sequence evolution and structural dynamics. Bioinformatics. 2014;30(18):2681-3.
12. Ruiz-Blanco YB, Paz W, Green J, Marrero-Ponce Y. ProtDCal: A program to compute general-purpose-numerical descriptors for sequences and 3D-structures of proteins. BMC Bioinf. 2015;16:162.
13. Mirdita M, von den Driesch L, Galiez C, Martin MJ, Soding J, Steinegger M. Uniclust databases of clustered and deeply annotated protein sequences and alignments. Nucleic Acids Res. 2017;45(D1):D170-D6.
14. Doerr S, Harvey MJ, Noe F, De Fabritiis G. HTMD: High-Throughput Molecular Dynamics for Molecular Discovery. J Chem Theory Comput. 2016;12(4):1845-52.
15. Krivak R, Hoksza D. P2Rank: machine learning based tool for rapid and accurate prediction of ligand binding sites from protein structure. J Cheminform. 2018;10(1):39.
16. Kaynak BT, Bahar I, Doruker P. Essential site scanning analysis: A new approach for detecting sites that modulate the dispersion of protein global motions. Comput Struct Biotechnol J. 2020;18:1577-86.
17. Lloyd SP. Least-squares quantization in PCM. IEEE Trans Inf Theory. 1982;28(2):129-37.
18. Rousseeuw PJ. Silhouettes - a Graphical Aid to the Interpretation and Validation of Cluster-Analysis. J Comput Appl Math. 1987;20:53-65.
19. Pedregosa F, Varoquaux G, Gramfort A, Michel V, Thirion B, Grisel O, et al. Scikit-learn: Machine Learning in Python. J Mach Learn Res. 2011;12:2825-30.
20. Comaniciu D, Meer P. Mean shift: A robust approach toward feature space analysis. IEEE Trans Pattern Anal Mach Intell. 2002;24(5):603-19.

21. Gullett JM, Cuypers MG, Grace CR, Pant S, Subramanian C, Tajkhorshid E, et al. Identification of Structural transitions in bacterial fatty acid binding proteins that permit ligand entry and exit at membranes. J Biol Chem. 2022:101676.
22. Sugiki T, Takeuchi K, Yamaji T, Takano T, Tokunaga Y, Kumagai K, et al. Structural basis for the Golgi association by the pleckstrin homology domain of the ceramide trafficking protein (CERT). J Biol Chem. 2012;287(40):33706-18.
23. Schrödinger, LLC. The PyMOL Molecular Graphics System, Version 2.0 2015.
