# Supplementary material for: DREAMM: a web-based server for drugging protein-membrane interfaces as a novel workflow for targeted drug design
Source: Bioinformatics. 2022 Nov 10;38(24):5449–51. doi: 10.1093/bioinformatics/btac680 (PMC9750117; doi:10.1093/bioinformatics/btac680)
Supplement: btac680_Supplementary_Data [file btac680_supplementary_data.zip › btac680_Supplementary_Data/OP-CBIO220690_AuthorCorr_AttachmentsFolder_manual[AU].pdf]

DREAMM

Drugging pRotein mEmbrAne Machine learning Method

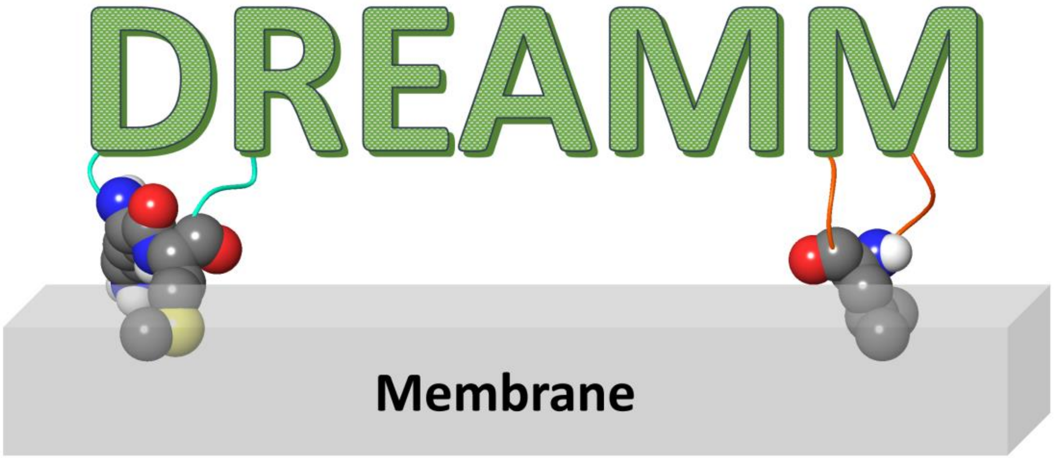

Alexios Chatzigoulas & Zoe Cournia

Biomedical Research Foundation

Academy of Athens

https://dreamm.ni4os.eu

Chatzigoulas A. and Cournia Z. Predicting protein-membrane interfaces of peripheral membrane proteins using ensemble machine learning. Brief Bioinform. 2022; 23(2), bbab518, 10.1093/bib/bbab518

Chatzigoulas A. and Cournia Z. DREAMM: A web-based server for drugging protein-membrane interfaces as a novel workflow for targeted drug design. Bioinformatics. 2022; btac680, 10.1093/bioinformatics/btac680

Contents

1. Methodology....3
2. Input....4
   a) Prediction of membrane-penetrating amino acids....4
      i. Insert PDB ID....4
      ii. Upload PDB file....4
   b) Prediction of membrane-penetrating amino acids and binding site prediction 5
      i. Insert NMR structure PDB ID....5
      ii. Upload PDB of a conformational ensemble....6
      iii. Generate a protein conformational ensemble with ExProSE....6
3. Output....8
   a) Display of the results....8
   b) Download results....9
4. Binding site clustering results interpretation....15
5. Using DREAMM with AlphaFold structures....17
6. Bibliography....19

1. Methodology

DREAMM implements machine learning to predict membrane-penetrating amino acids of peripheral membrane proteins and predicts binding sites near the predicted membrane-penetrating amino acids in protein conformational ensembles. To predict the membrane-penetrating amino acids, a novel ensemble machine learning classifier model was trained using experimental data retrieved from an extensive literature search (1).

When a PDB structure is entered in DREAMM, firstly it is prepared with HTMD (2) and then the feature extraction begins, generating various physicochemical and biochemical features. These features include the secondary structure definition using DSSP (3), the solvent-accessible surface area using FreeSASA (4), the amino acid and Cα depth using MSMS (5), the Wimley-White whole-residue interface, and octanol hydrophobicity scales (6, 7), the charges using PDB2PQR (8, 9), the conservation score using HHblits (10), the squared fluctuations using PRODY (11, 12), the number of nearby amino acids, and others. Furthermore, to consider the surrounding amino acid properties of each amino acid, the mean values of the aforementioned features are calculated, for each amino acid and the amino acids lying at a distance of Cα - Cα 7 Å. In addition, the ProtDCal tool is implemented (13), which calculates numerous thermodynamics, topographic, and property-based features.

When the feature extraction is completed, the ensemble classifier model predicts the membrane-penetrating amino acids. To reduce the false positive non-hydrophobic amino acids, DREAMM labels as membrane-penetrating only the non-hydrophobic amino acids that lie at a COM-COM distance of 14 Å from at least one of the predicted hydrophobic amino acids. The results are displayed on the web server and visualized with JSmol (14, 15).

Moreover, the user may choose to search for binding sites in the vicinity of the predicted membrane-penetrating interface. To take into account the dynamic nature of proteins, DREAMM searches for binding sites in conformational ensembles. After the user inputs the protein conformational ensemble, the P2Rank stand-alone open-source software (16) is used to predict binding sites in each protein conformation, separately. The PyMOL (17) scripts that produce 3D visualizations generated from P2Rank are automatically modified to display the predicted membrane-penetrating amino acids in purple and the binding sites within a distance of 5 Å from the closest atom of the predicted membrane-penetrating amino acids, which are then clustered based on their center coordinates. A unique identifier (UID) is assigned to each job and a unique URL for each job is provided to the user. The final results can be downloaded through the web server. For more details regarding the methodology please refer to Ref. (1).

2. Input

a) Prediction of membrane-penetrating amino acids

The users interested to predict membrane-penetrating amino acids proceed with the calculation by uploading the PDB file and do not check the binding site prediction box.

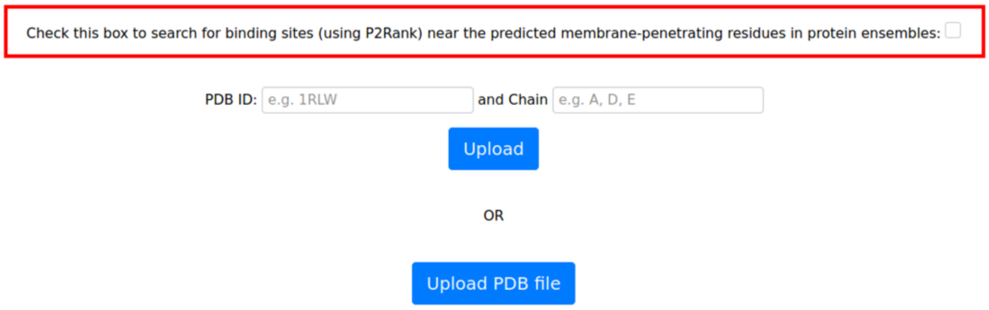

i. Insert PDB ID

Users input the PDB ID and the chains and hit the “Upload” button. If the “Chain” field is empty the whole structure will be used to predict membrane-penetrating amino acids.

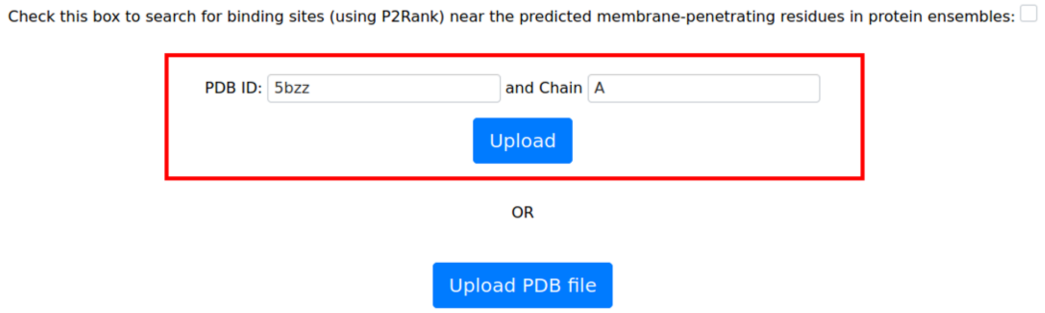

ii. Upload PDB file

Users may also choose to upload their own protein structure by hitting the “Upload PDB file” button. Once the user uploads the structure the prediction starts automatically.

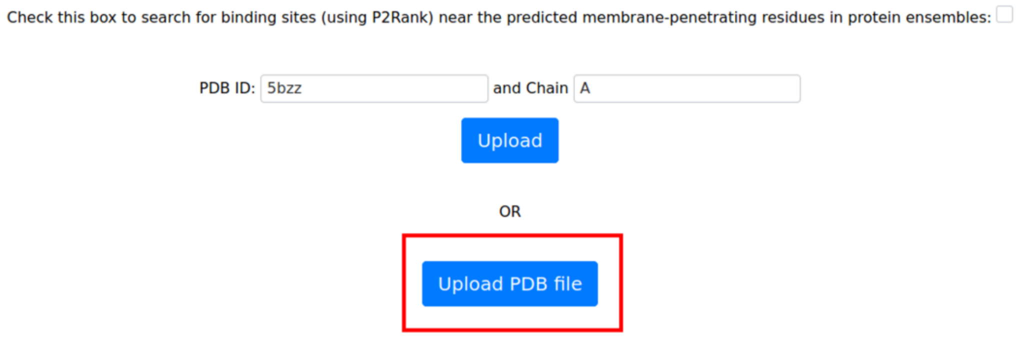

b) Prediction of membrane-penetrating amino acids and binding site prediction

In case users want to search for binding sites near the predicted membrane-penetrating amino acids, they must check the appropriate box. Once it is checked, new options will appear. The binding site prediction is performed in protein conformational ensembles and the membrane-penetrating amino acids prediction will be performed in the first model of the ensemble.

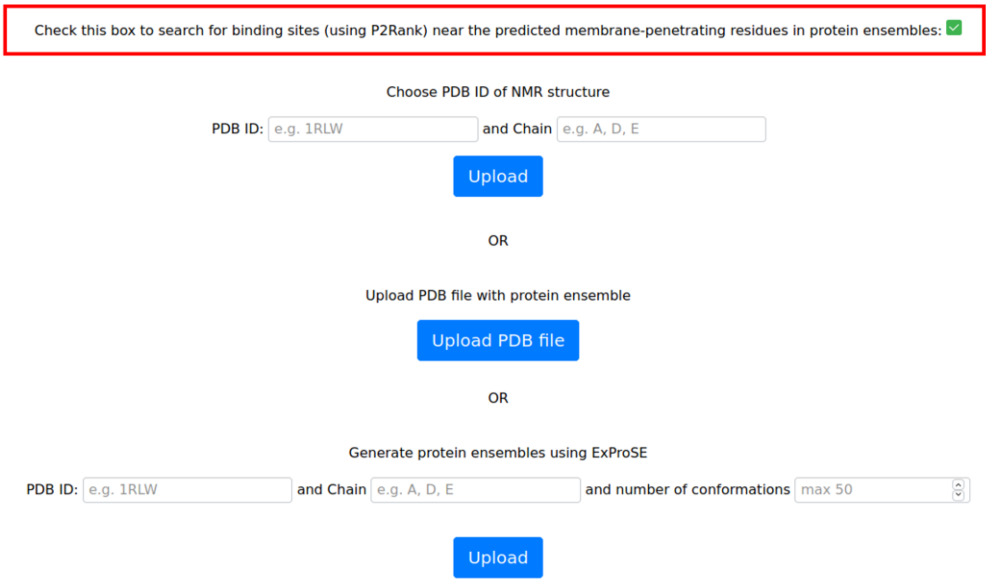

i. Insert NMR structure PDB ID

The first option is to input the PDB ID of an NMR structure, choose the chains, and hit the “Upload” button. If the “Chain” field is empty, the whole structure will be used to predict membrane-penetrating amino acids and binding sites. If the PDB file is not an NMR structure, DREAMM will proceed with the calculation and the predictions even if it is an X-ray structure.

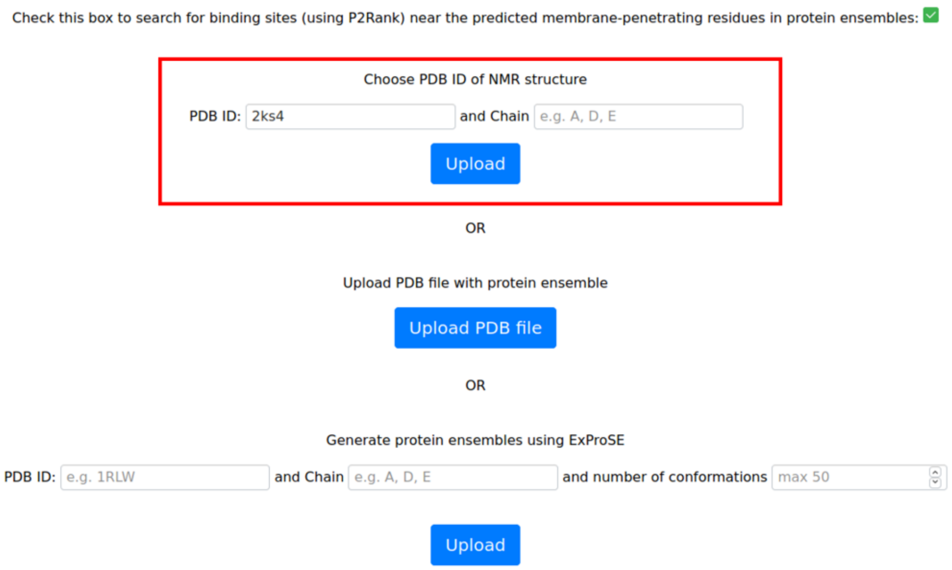

ii. Upload PDB of a conformational ensemble

Users may also upload their own protein conformational ensemble by hitting the "Upload PDB file" button and selecting their file. Once the users upload their structure, the prediction starts automatically. If the PDB contains only one model, DREAMM will proceed with the calculation in this model.

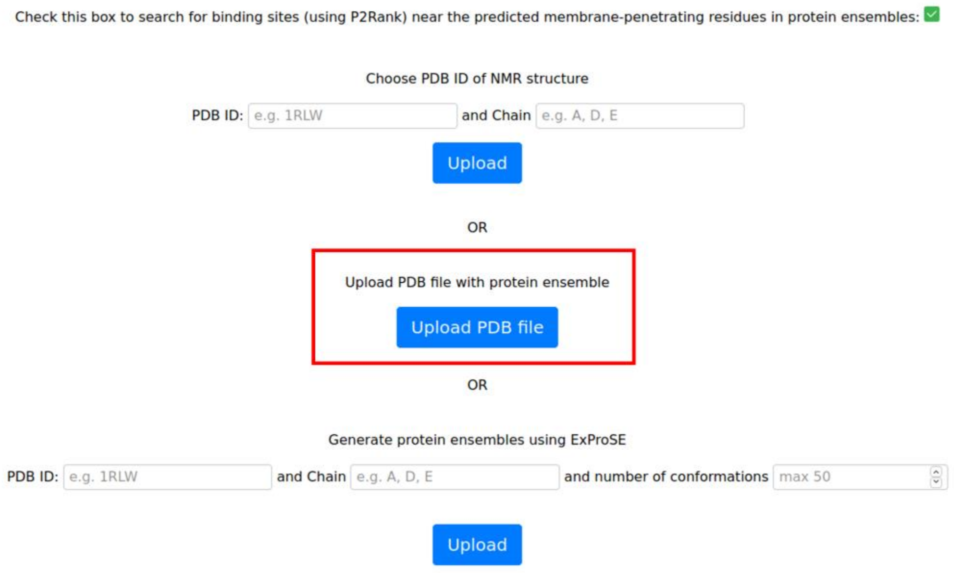

Important note: The protein conformational ensemble must be in a PDB file format with the models divided by MODEL / ENDMDL records!

iii. Generate a protein conformational ensemble with ExProSE

In case NMR structures or a protein conformational ensemble are not available, the users may create a protein conformational ensemble using ExProSE. The users input the PDB ID, choose the chains and the number of additional conformations to generate, and hit the "Upload" button. If the "Chain" field is empty, the whole structure will be used to predict membrane-penetrating amino acids and generate conformational ensembles. The limit for the number of conformations that can be generated is 50. The binding site prediction is also performed in the initial structure, so if the user chooses to generate 20 conformations with ExProSE, the output will contain 21 protein conformations. The membrane-penetrating amino acid prediction will be carried out in the initial PDB and not in one of the generated conformations.

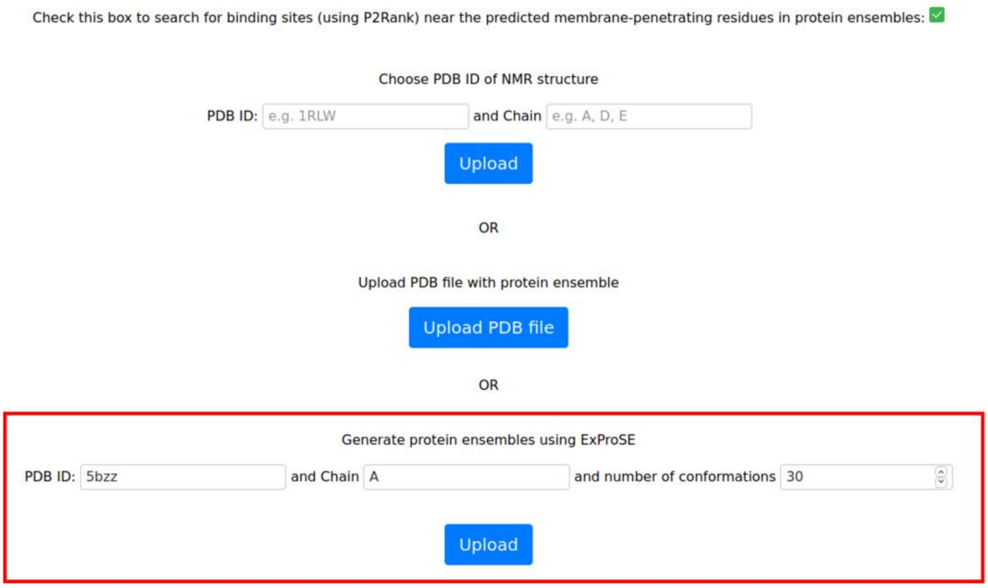

Important note: ExProSE is programmed to generate conformational ensembles between apo and holo structures, but it can also generate protein conformations with one structure as input. For the convenience of the users, we accept only one structure as input. To generate protein conformations, we apply the default ExProSE settings with a tolerance weighting factor (weighting of constraint tolerances for interactions) $W_B = 0.4$ to avoid large conformational changes.

3. Output

a) Display of the results

After the calculation finishes, the predicted membrane-penetrating amino acids are displayed in the format:

The residues: "Chain name" "resid" "resid" … "resid" "Chain name" "resid" "resid" … "resid" … are predicted to insert the membrane

The protein is visualized with JSmol (14, 15) in secondary structure representation and the membrane-penetrating amino acids in CPK.

The residues: A 42 263 264 are predicted to insert the membrane.

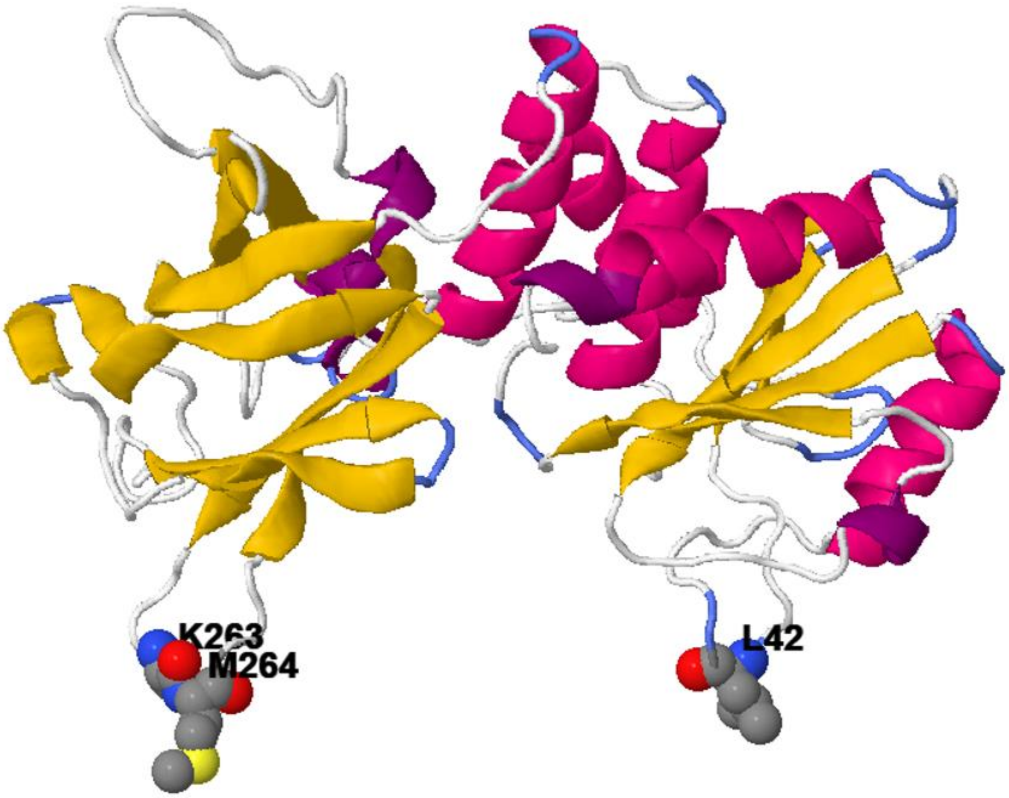

b) Download results

Users may download the results by hitting the “Download Results” button or return to the main page to perform further predictions by hitting the “Return” button. The buttons are placed just below the JSmol visualization.

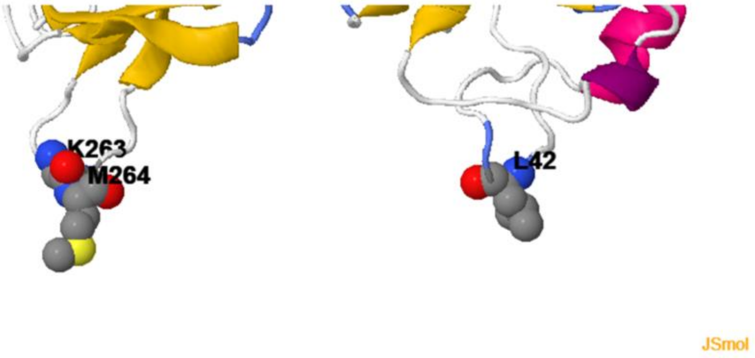

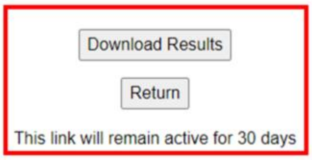

The downloaded results depend on whether the user chooses to predict binding sites or not.

If the user does **not** choose to predict binding sites, then a csv file will be downloaded including the membrane penetrating amino acid predictions. Specifically, the csv file includes the chain name, the residue number (“resnum” column), the one-code amino acid letter, and the “broken_chain” column, which indicates whether the prediction is near the N- or C-termini, or near missing loops, which might generate a false positive prediction (0 denotes **not** being in the N- or C-termini or near missing loops, and 1 denotes amino acids that are found in these regions).

chain	resnum	Amino acid	broken_chain
A	42	L	0
A	263	K	0
A	264	M	0

If the user chooses to predict binding sites, a zip file will be downloaded with a folder containing the following files in the following directory structure:

```
|-- "PDB"_"UID".csv
|-- input.pdb
|-- pcs
|   |-- evals_spread.tsv
|   |-- pc_1_2.png
|   |-- pc_1_3.png
|   |-- pc_2_3.png
|   |-- pcs.tsv
|   `-- pcs_input.tsv
|-- pdbs
|   |-- "PDB"_"UID"_0.pdb
|   |-- "PDB"_"UID"_1.pdb
|   |-- …
|   `-- prepared
|       |-- "PDB"_"UID"_0_fixed.pdb
|       |-- "PDB"_"UID"_1_fixed.pdb
|       |-- …
|       |-- fixed.txt
|       `-- pockets
|           |-- "PDB"_"UID"_0_fixed.pdb_predictions.csv
|           |-- "PDB"_"UID"_0_fixed.pdb_residues.csv
|           |-- "PDB"_"UID"_1_fixed.pdb_predictions.csv
|           |-- "PDB"_"UID"_1_fixed.pdb_residues.csv
|           |-- …
|           |-- params.txt
|           |-- results
|           |   |-- "PDB"_"UID".csv
|           |   |-- "PDB"_"UID".xlsx
|           |   |-- "PDB"_"UID"_clustered.csv
|           |   |-- "PDB"_"UID"_clustered.xlsx
|           |   `-- results.txt
|           |-- run.log
|           `-- visualizations
|               |-- "PDB"_"UID"_0_fixed.pdb.pml
|               |-- "PDB"_"UID"_1_fixed.pdb.pml
|               |-- …
|               `-- data
|                   |-- "PDB"_"UID"_0_fixed.pdb_points.pdb.gz
|                   |-- "PDB"_"UID"_1_fixed.pdb_points.pdb.gz
|                   |-- …
|-- pymol
|   |-- view_pc_1.pml
|   |-- view_pc_2.pml
|   |-- view_pc_3.pml
```

```
|   `-- view_pc_4.pml
|-- rmsds.tsv
|-- rmsfs.png
|-- rmsfs.tsv
`-- spe_scores.tsv
```

The directories and files are explained below:

"PDB"_"UID".csv: The abovementioned .csv file with the membrane-penetrating amino acid predictions.

input.pdb: It is output only if ExProSE is used. The input PDB file. For more information, please refer to https://github.com/jgreener64/ProteinEnsembles.jl.

rmsds.tsv: It is output only if ExProSE is used. Root-mean-square deviations (RMSDs) of generated structures to the input structures. Line *n* corresponds to structure *n*. For more information, please refer to https://github.com/jgreener64/ProteinEnsembles.jl.

rmsfs.png and rmsfs.tsv: It is output only if ExProSE is used. Root-mean-square fluctuations (RMSFs) of each residue over the ensemble of generated structures, and the corresponding plot. Line *n* corresponds to residue index *n*. For more information, please refer to https://github.com/jgreener64/ProteinEnsembles.jl.

spe_scores.tsv: It is output only if ExProSE is used. Stochastic proximity embedding (SPE) error scores of generated structures. Line *n* corresponds to structure *n*. For more information, please refer to https://github.com/jgreener64/ProteinEnsembles.jl.

pcs: It is output only if ExProSE is used. It contains the projections onto the principal components (PCs) from the principal component analysis of the generated structures. For more information, about the files in this directory please refer to https://github.com/jgreener64/ProteinEnsembles.jl.

pymol: It is output only if ExProSE is used. PyMOL scripts to view PCs on input.pdb, e.g., run pymol input.pdb pymol/view_pc_1.pml. For more information, please refer to https://github.com/jgreener64/ProteinEnsembles.jl.

pdbs/"PDB"_"UID"_#.pdb: The input structure and the generated structures produced by ExProSE. The input structure is the "PDB"_"UID"_0.pdb and the generated structures are the "PDB"_"UID"_1.pdb, "PDB"_"UID"_2.pdb, etc. Note that the numbering in the files starts from zero.

pdbs/prepared/"PDB"_"UID"_#_fixed.pdb: The prepared structures using HTMD. The "PDB"_"UID"_0_fixed.pdb is the prepared structure of the first model in the conformational ensemble, the "PDB"_"UID"_1_fixed.pdb is the prepared structure of the second model in the conformational ensemble, etc. Note that the numbering in the files starts from zero.

pdbs/prepared/fixed.txt: This file contains a list of the names of the prepared structures.

pdbs/prepared/pockets/"PDB"_"UID"_#_fixed.pdb_predictions.csv: Produced by P2Rank. It contains an ordered list of predicted pockets for protein conformation #, their scores, the coordinates of their centers together with a list of adjacent residues, a list of adjacent protein surface atoms, and a calibrated probability of being a ligand-binding site. Note that the numbering in the files starts from zero. For more information, please refer to https://github.com/rdk/p2rank.

pdbs/prepared/pockets/"PDB"_"UID"_#_fixed.pdb_residues.csv: Produced by P2Rank. It contains a list of all residues from the input protein conformation #, with their scores, mapping to predicted pockets, and a calibrated probability of being a ligand-binding residue. Note that the numbering in the files starts from zero. For more information, please refer to https://github.com/rdk/p2rank.

pdbs/prepared/pockets/params.txt: The input parameters of P2Rank. For more information, please refer to https://github.com/rdk/p2rank.

pdbs/prepared/pockets/run.log: The run log of P2Rank.

pdbs/prepared/pockets/visualizations/"PDB"_"UID"_#_fixed.pdb.pml: The PyMOL visualization of protein conformation # showing the predicted membrane-penetrating amino acids with purple, the protein in secondary structure representation, and the predicted binding sites within a distance of 5 Å from the predicted membrane-penetrating amino acids. Note that the numbering in the files starts from zero.

pdbs/prepared/pockets/visualizations/data/"PDB"_"UID"_#_fixed.pdb._points.pdb.gz: Produced by P2Rank. Coordinates of the Solvent Accessible Surface (SAS) points for protein conformation #. The "Residue sequence number" (23-26) of the HETATM record corresponds to the rank of the corresponding pocket (points with the value 0 do not belong to any pocket). Note that the numbering in the files starts from zero. For more information, please refer to https://github.com/rdk/p2rank.

pdbs/prepared/pockets/results/: Contains five files: The "PDB"_"UID" file in two file formats (csv and xlsx), the "PDB"_"UID"_clustered file in two file formats (csv and xlsx), and the results.txt file.

Specifically, the "PDB"_"UID".csv and "PDB"_"UID".xlsx files contain all binding sites within a distance of 5 Å from the predicted membrane-penetrating amino acids in all conformations. Each row represents a binding site and the columns show:

1. the predicted membrane-penetrating amino acid near the binding site ("residue" column),
2. the smallest distance between the predicted membrane-penetrating amino acid and the binding site in Å ("dist_surf" column),
3. the P2Rank ranking of the binding site ("site rank" column),

4. the P2Rank score of the binding site ("score" column),
5. the distance between the predicted membrane-penetrating amino acid and the binding site center in Å ("dist_center" column),
6. the protein conformation ("conformation" column),
7. the residue IDs near the binding pocket surface ("res_id" column),
8. the atom IDs near the binding pocket surface ("surf_atoms" column), and
9. the binding site center coordinates in Å ("center_x", "center_y", and "center_z" columns).

The "PDB"_"UID"_clustered.csv and .xlsx files contain the binding site clustering results. Each row represents a binding site within a distance of 5 Å from the predicted membrane-penetrating amino acids and the columns show:

1. Binding site number.
2. The protein conformations where the binding site is found and their PyMOL pocket number ("conformation_#_pocket_#" column),
3. The total number of protein conformations that the binding site is found ("total_conformations" column),
4. the predicted membrane-penetrating amino acids near the binding site ("membrane-penetrating_residues" column),
5. the mean and std of the smallest distance between the predicted membrane-penetrating amino acid and the binding site among the conformations that the binding site is found in Å ("dist_surf_mean" and "dist_surf_std" columns),
6. the mean and std of the distance between the predicted membrane-penetrating amino acid and the binding site center among the conformations that the binding site is found in Å ("dist_center_mean" and "dist_center_std" columns),
7. the highest P2Rank score of the binding site among the conformations that the binding site is found ("highest_score" column),
8. the conformation with the highest P2Rank score ("highest_score_conf" column),
9. the mean and std of the P2Rank ranking of the binding site among the conformations that the binding site is found ("site_rank_mean" and "site_rank_std" columns),
10. the mean and std of the binding site center coordinates among the conformations that the binding site is found in Å ("center_x_mean", "center_x_std", "center_y_mean", "center_y_std", "center_z_mean" and "center_z_std" columns),
11. the mean and std of the binding site conservation score among the conformations that the binding site is found ("conservation_mean" and "conservation_std" columns), and
12. the mean and std of the binding site ESSA *z*-score among the conformations that the binding site is found ("ESSA_ mean" and "ESSA_std" columns).

The results.txt file contains the summarized binding site clustering results. The first line mentions the number of binding sites in the vicinity of the predicted membrane-penetrating amino acids. Then, each line represents a binding site reporting on:

1. the number of protein conformations where the binding site is found,
2. the predicted membrane-penetrating amino acids near the binding site,
3. the highest P2Rank score of the binding site among the conformations that the binding site is found,
4. the mean ± std of the smallest distance in Å between the predicted membrane-penetrating amino acid and the binding site among the conformations that the binding site is found,
5. the mean ± std of the binding site center coordinates in Å among the conformations that the binding site is found,
6. the mean ± std of the binding site conservation score among the conformations that the binding site is found, and
7. the mean and std of the binding site ESSA *z*-score among the conformations that the binding site is found.

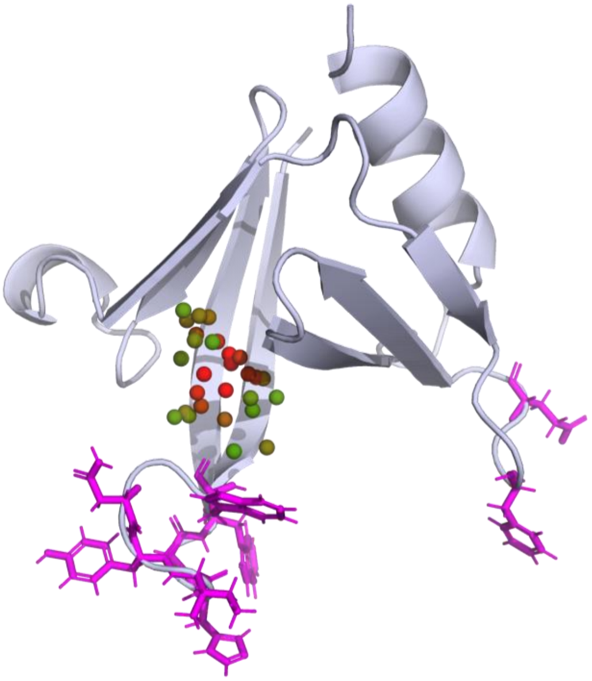

As an example, we report the first model from the NMR structural ensemble 2RSG where only one of the three binding sites predicted by P2Rank is displayed in PyMOL, as it is the only one at a distance of 5 Å from the predicted membrane-penetrating amino acids.

Note: If users are interested in visualizing the rest of the discovered binding pockets by P2Rank, all pockets are stored in the generated PyMOL session.

4. Binding site clustering results interpretation

As a use case, we utilize the prothrombin protein with PDB ID: 5EDM and generate 20 additional conformations using ExProSE. The predicted membrane-penetrating amino acids are displayed, visualized, and can be also downloaded from the DREAMM web server. DREAMM assigns a unique ID (UID) in each job, for example, 20220419145002. The prefix of the downloaded files is the "PDB"_"UID"; in our case, it is the 5EDM_20220419145002. The results.txt file contains information about 8 discovered binding sites located at the predicted protein-membrane interfaces (the results may differ due to the statistical nature of ExProSE). For each binding site DREAMM provides the binding site statistics in the following format (the binding sites are ranked based on the P2Rank score):

Binding site 1 is found in 21/21 conformations, near the predicted membrane-penetrating amino acids ['A_398', 'A_458'], with highest P2Rank score 29.3414, average distance from the binding site surface 2.6 ± 1.31, average distance from the binding site center 9.4 ± 0.44, average conservation score 0.31 ± 0.04, and average ESSA score 0.31 ± 0.29.

Binding site 2 is found in 20/21 conformations, near the predicted membrane-penetrating amino acids ['A_458'], with highest P2Rank score 26.0233, average distance from the binding site surface 1.33 ± 1.8, average distance from the binding site center 9.29 ± 2.08, and average conservation score 0.26 ± 0.02, and average ESSA score 0.2 ± 0.31.

…

Binding site 8 is found in 10/21 conformations, near the predicted membrane-penetrating amino acids ['A_93'], with highest P2Rank score 2.1478, average distance from the binding site surface 0.72 ± 1.52, average distance from the binding site center 3.83 ± 1.9, average conservation score 0.24 ± 0.03, and average ESSA score -0.99 ± 0.14.

More details about clustering results are provided in the 5EDM_20220419145002_clustered.csv (or .xlsx) file. In this file, the binding sites are ranked based on their highest P2Rank score. For each binding site, a list of all conformations that this binding site is found along with its PyMOL pocket number can be located in the "conformation_#_pocket_#" column in the following format:

['5EDM_20220419145002_0_fixed.pdb_pocket_1'

'5EDM _20220419145002_10_fixed.pdb_pocket_1'

…

'5EDM_20220419145002_9_fixed.pdb_pocket_2']

For example, if the user wants to visualize this binding site in the 10th protein conformation, the user has to open the 5EDM_20220419145002_9_fixed.pdb.pml file (note that the numbering in the files starts from zero) from the "pdbs/prepared/pockets/visualizations" folder in PyMOL and select pocket 2.

If the user wants to proceed with structure-based drug design in the binding site with the highest P2Rank score, which is usually the consensus binding site, the user selects one of the conformations of "Binding site 1". The user may locate the protein conformation with the highest P2Rank score in the "highest_score_conf" column. However, the user may want to target a binding site that is near a specific predicted membrane-penetrating amino acid, for example, Y93. In the "membrane-penetrating_residues" column of 5EDM_20220419145002_clustered.csv (or .xlsx) file, the user can find the predicted membrane-penetrating amino acids, which are adjacent to each binding site. To conclude, based on the predicted membrane-penetrating amino acids, the user can choose the corresponding binding site, from the "conformation_#_pocket_#" column the user can locate the conformations where this binding site is located along with its PyMOL pocket number, and in the "highest_score_conf" column the user can locate the conformation with the highest P2Rank score.

5. Using DREAMM with AlphaFold structures

With the recent advancements in protein structure predictions, i.e., AlphaFold2 (18) and RoseTTAFold (19), the structure of unresolved proteins can be predicted with high accuracy; but in many cases, these models fail to fold the N- or C-terminus or various protein segments. It is thus recommended to remove these regions, i.e., amino acids with a confidence predicted local-distance difference test score (pLDDT) < 70 before applying DREAMM because these unfolded regions affect the prediction accuracy.

For example, in the interleukin-22 receptor subunit alpha-1 prediction of AlphaFold2, https://alphafold.ebi.ac.uk/entry/Q8N6P7,

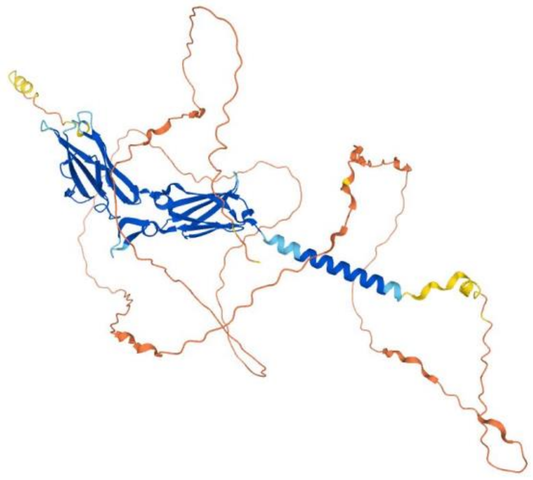

the unfolded regions affect DREAMM predictions due to high solvent exposure,

The residues: A 1* 2* 3* 4 5 7 8 10 13 14 16 227 229 234 235 236 237 239 240 241 242 243 244 245 246 247 248 249 251 252 253 254 256 264 268 269 270 271 272 273 275 276 277 278 282 283 284 286 287 288 289 304 305 306 310 327 332 334 341 342 345 346 349 351 353 361 362 366 368 374 375 376 377 382 385 390 392 399 403 405 406 407 419 424 425 426 441 442 445 447 450 453 494 498 499 504 507 509 513 530 532 533 535 536 537 538 539 546 559 562 563 564 566 572* are predicted to insert the membrane.

Residues near the N- or C- terminal, or near missing loops are denoted with * and might be false positives

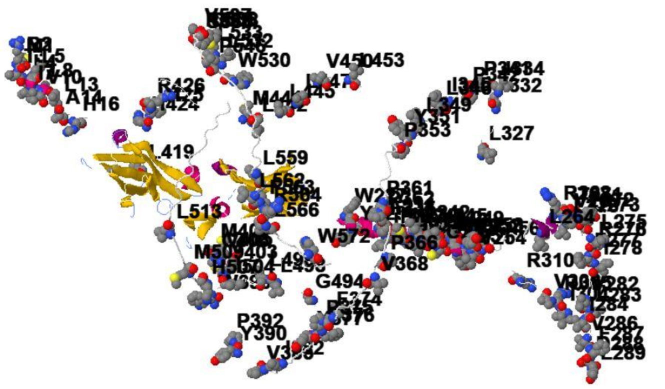

but, if we remove the amino acids with a confidence score (pLDDT) less than 70,

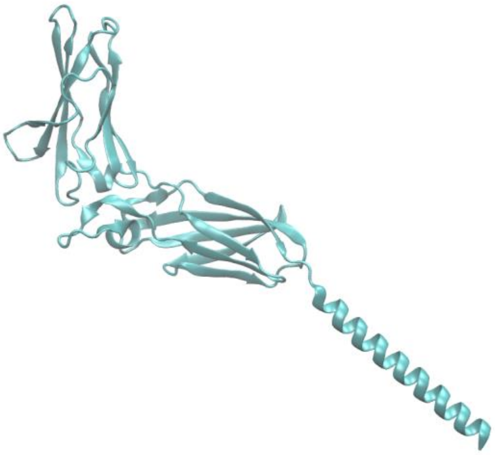

DREAMM predictions are accurate, correctly predicting the α helix 225-255 that inserts in the membrane.

The residues: A 225 227 229 231 233 234 235 236 237 239 240 241 242 243 244 245 246 247 248 249 251 252 253* 254* 255* are predicted to insert the membrane.

Residues near the N- or C- terminal, or near missing loops are denoted with * and might be false positives

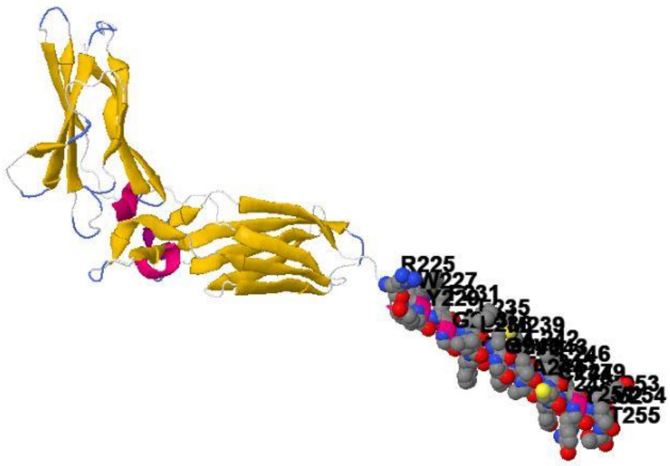

If you encounter any problems, please feel free to contact us at: a.chatzigoulas@gmail.com

6. Bibliography

1. Chatzigoulas A, Cournia Z. Predicting protein–membrane interfaces of peripheral membrane proteins using ensemble machine learning. Brief Bioinform. 2022;23(2):bbab518.
2. Doerr S, Harvey MJ, Noe F, De Fabritiis G. HTMD: High-Throughput Molecular Dynamics for Molecular Discovery. J Chem Theory Comput. 2016;12(4):1845-52.
3. Kabsch W, Sander C. Dictionary of protein secondary structure: pattern recognition of hydrogen-bonded and geometrical features. Biopolymers. 1983;22(12):2577-637.
4. Mitternacht S. FreeSASA: An open source C library for solvent accessible surface area calculations. F1000Res. 2016;5:189.
5. Sanner MF, Olson AJ, Spehner JC. Reduced surface: an efficient way to compute molecular surfaces. Biopolymers. 1996;38(3):305-20.
6. Wimley WC, White SH. Experimentally determined hydrophobicity scale for proteins at membrane interfaces. Nat Struct Mol Biol. 1996;3(10):842-8.
7. Wimley WC, Creamer TP, White SH. Solvation energies of amino acid side chains and backbone in a family of host-guest pentapeptides. Biochemistry. 1996;35(16):5109-24.
8. Dolinsky TJ, Nielsen JE, McCammon JA, Baker NA. PDB2PQR: an automated pipeline for the setup of Poisson-Boltzmann electrostatics calculations. Nucleic Acids Res. 2004;32:W665-7.
9. Dolinsky TJ, Czodrowski P, Li H, Nielsen JE, Jensen JH, Klebe G, et al. PDB2PQR: expanding and upgrading automated preparation of biomolecular structures for molecular simulations. Nucleic Acids Res. 2007;35:W522-5.
10. Remmert M, Biegert A, Hauser A, Soding J. HHblits: lightning-fast iterative protein sequence searching by HMM-HMM alignment. Nat Methods. 2011;9(2):173-5.
11. Bakan A, Meireles LM, Bahar I. ProDy: protein dynamics inferred from theory and experiments. Bioinformatics. 2011;27(11):1575-7.
12. Bakan A, Dutta A, Mao W, Liu Y, Chennubhotla C, Lezon TR, et al. Evol and ProDy for bridging protein sequence evolution and structural dynamics. Bioinformatics. 2014;30(18):2681-3.
13. Ruiz-Blanco YB, Paz W, Green J, Marrero-Ponce Y. ProtDCal: A program to compute general-purpose-numerical descriptors for sequences and 3D-structures of proteins. BMC Bioinform. 2015;16:162.
14. Hanson RM. Jmol–a paradigm shift in crystallographic visualization. J Appl Crystallogr. 2010;43(5):1250-60.
15. Hanson RM, Prilusky J, Renjian Z, Nakane T, Sussman JL. JSmol and the Next-Generation Web-Based Representation of 3D Molecular Structure as Applied to Proteopedia. Isr J Chem. 2013;53(3-4):207-16.
16. Krivak R, Hoksza D. P2Rank: machine learning based tool for rapid and accurate prediction of ligand binding sites from protein structure. J Cheminform. 2018;10(1):39.
17. Schrödinger, LLC. The PyMOL Molecular Graphics System, Version 2.0 2015.
18. Jumper J, Evans R, Pritzel A, Green T, Figurnov M, Ronneberger O, et al. Highly accurate protein structure prediction with AlphaFold. Nature. 2021;596(7873):583-9.
19. Baek M, DiMaio F, Anishchenko I, Dauparas J, Ovchinnikov S, Lee GR, et al. Accurate prediction of protein structures and interactions using a three-track neural network. Science. 2021;373(6557):871-6.
